# Risk of seizures after immunization in children with epilepsy: a risk interval analysis

**DOI:** 10.1186/s12887-018-1112-0

**Published:** 2018-04-11

**Authors:** Karina A. Top, Paula Brna, Lingyun Ye, Bruce Smith

**Affiliations:** 1 Department of Pediatrics, Dalhousie University, Halifax, NS Canada; 2 Department of Community Health & Epidemiology, Dalhousie University, Halifax, NS Canada; 3 Canadian Center for Vaccinology, IWK Health Centre, 4th Floor Goldbloom RCC Pavilion, 5850/5980 University Ave, Halifax, NS B3K 6R8 Canada; 4 Department of Mathematics and Statistics, Dalhousie University, Halifax, NS Canada

**Keywords:** Adverse event following immunization, Epilepsy, Seizure, Vaccination, Vaccine safety

## Abstract

**Background:**

In children with epilepsy, fever and infection can trigger seizures. Immunization can also induce inflammation and fever, which could theoretically trigger a seizure. The risk of seizure after immunization in children with pre-existing epilepsy is not known. The study objective was to determine the risk of medically attended seizure after immunization in children with epilepsy < 7 years of age.

**Methods:**

We conducted a retrospective study of children < 7 years of age with epilepsy in Nova Scotia, Canada from 2010 to 2014. Hospitalizations, emergency visits, unscheduled clinic visits, and telephone calls for seizures were extracted from medical records. Immunization records were obtained from family physicians and Public Health with informed consent. We conducted a risk interval analysis to estimate the relative risk (RR) of seizure during risk periods 0–14, 0–2, and 5–14 days post-immunization versus a control period 21–83 days post-immunization.

**Results:**

There were 302 children with epilepsy who were eligible for the study. Immunization records were retrieved on 147 patients (49%), of whom 80 (54%) had one or more immunizations between the epilepsy diagnosis date and age 7 years. These 80 children had 161 immunization visits and 197 medically attended seizures. Children with immunizations had more seizures than either those with no immunizations or those with no records (mean 2.5 versus 0.7 versus 0.9, *p* < 0.001). The risk of medically attended seizure was not increased 0–14 days after any vaccine (RR = 1.1, 95% confidence interval (CI): 0.5–2.8) or 0–2 days after inactivated vaccines (RR = 0.9, 95% CI: 0.1–7.1) versus 21–83 days post-immunization. No seizure events occurred 5–14 days after live vaccines.

**Conclusions:**

Children with epilepsy do not appear to be at increased risk of medically attended seizure following immunization. These findings suggest that immunization is safe in children with epilepsy, with benefits outweighing risks.

**Electronic supplementary material:**

The online version of this article (10.1186/s12887-018-1112-0) contains supplementary material, which is available to authorized users.

## Background

Immunization programs are among the most effective public health interventions ever developed. [1] However, as the incidence of vaccine-preventable infections has decreased, healthcare professionals and the public have become increasingly concerned about the risk of adverse events following immunization (AEFIs). [2] Neurological events are among the most serious AEFIs, frequently resulting in emergency visits or hospitalization. Seizure is both the most frequent neurological AEFI reported and the most common neurological disorder in children. [3] Up to 10% of children will experience a seizure by their 16th birthday, and approximately 0.5% of children have epilepsy, making it one of the more common chronic diseases in children. [4–6]

Certain vaccines [e.g., measles-mumps-rubella (MMR) vaccine, trivalent inactivated influenza vaccine] have been associated with febrile seizures in young children. [7–11] The timing of febrile seizures after immunization coincides with the peak inflammatory response. Inactivated vaccines such as inactivated influenza vaccine stimulate an immune response within hours, inducing fever (and febrile seizure) within 1–2 days after immunization. [7, 8] After live vaccines (e.g., MMR), the risk of febrile seizure is delayed until 5–14 days post-immunization, after the attenuated virus has reached sufficient levels in the bloodstream to be detected by the immune system. [9, 10] In contrast, immunizations have not been associated with afebrile seizures in large cohort studies, and several studies and reviews concluded that immunizations do not trigger the onset of epilepsy [9, 12–15]. However, the risk of seizure after routine immunizations in children with pre-existing epilepsy has not been systematically evaluated. Despite limited direct evidence of vaccine safety in this population, current practice guidelines recommend that children with epilepsy follow the routine immunization schedule. [16]

The study objective was to estimate the relative risk of medically attended seizure after immunization among children less than 7 years of age with a diagnosis of epilepsy. Evidence of the safety of immunization in these children would reassure parents and healthcare providers concerned about the risk of immunization-induced seizures.

## Methods

### Study design and subjects

We conducted a retrospective cohort study of children diagnosed with epilepsy before 7 years of age who lived in Nova Scotia, Canada and were followed by the IWK Health Centre Neurology Service between January 2010 and December 2014. The IWK Health Centre is the only pediatric tertiary care center in the province of Nova Scotia (2016 population: 949,500 [17]) and the IWK Neurology Service follows approximately 95% of children with epilepsy in the province. [18] Children were eligible for the study if they had a neurologist diagnosis of epilepsy before their seventh birthday, were seen by a neurologist based at the IWK between 2010 and 2014, and were a resident of Nova Scotia on the date of the first clinic visit. Children with a history of seizures that occurred exclusively during the neonatal period or only with fever (i.e., febrile seizures) were excluded.

Children with epilepsy were identified by screening medical records of patients seen in the IWK Neurology Clinic. Caregivers of children who met the inclusion criteria were contacted initially via mail, followed by a telephone call inviting them to participate in the study by providing their verbal and/or written consent to release their child’s immunization records. The study team attempted to contact caregivers by telephone up to five times, calling at different times of the day and leaving up to two messages.

### Data sources

Healthcare encounters for new or escalating seizures, including hospitalizations, emergency visits, unscheduled outpatient visits and telephone calls to the neurologist were extracted from the electronic medical record and Neurology clinic chart. Families of children with epilepsy are instructed to call the on-call neurologist to notify them of seizure events and request advice; details of these calls are recorded in the clinic chart. Patient demographics (sex, month/year of birth), age at epilepsy diagnosis, epilepsy type [severe epilepsy (defined as severe myoclonic epilepsy of infancy/Dravet syndrome, infantile spasms, and Lennox Gastaux syndrome), partial (focal) epilepsy, idiopathic generalized epilepsy (including absence epilepsy), unclassified epilepsy], antiepileptic therapy, and dates of healthcare encounters were collected.

Immunization records were retrieved from the child’s family physician and/or Nova Scotia Public Health Services. In Nova Scotia, at least 80% of immunizations among children < 7 years of age are administered by family physicians who are required to submit a notification form to Public Health with the details of the vaccines administered. Approximately 20% of immunizations are administered by public health nurses. To ensure data completeness, immunization dates and vaccines administered were extracted from both sources whenever possible.

### Statistical analysis

The primary exposure was any immunization received between the date of epilepsy diagnosis and the seventh birthday. The primary outcome was seizure requiring medical attention between the date of epilepsy diagnosis and the seventh birthday. For patients with > 1 healthcare encounter within 24 h, the highest acuity healthcare encounter was included in the analysis. Age at diagnosis, epilepsy type, and number of seizure events were compared among children with and without immunization visits and those whose records were not available (includes children who did not consent and those who consented but whose records were not available from either Public Health or the primary care physician). Categorical variables were assessed by χ^2^ test or Fisher’s exact tests for expected cell sizes < 5 and continuous variables were assessed by ANOVA. Statistical significance was defined as *p* < 0.05.

The risk of seizure-related healthcare encounter after immunization was assessed in a risk interval analysis using unconditional Poisson regression models. [19] This approach compares the rate of adverse events during a “risk period” when an adverse reaction to the vaccine is biologically plausible, to the rate during a “control period” remote from immunization when the vaccine cannot be implicated. Assuming causality, the relative risk (RR) of seizure during the risk versus control windows corresponds to the RR of vaccine-attributable events. This approach implicitly adjusts for fixed confounders (e.g., epilepsy type). The RR of seizure requiring medical attention was assessed during five risk periods: 0–14 days after any vaccine, 0–2 days after inactivated vaccine, 0–3 days after inactivated vaccine, 7–10 days after live vaccine, and 5–14 days after a live vaccine. A control period of 21–83 days post-immunization was used for all analyses. A control period of 7–83 days pre-immunization was used in a sensitivity analysis. Days 15–20 represented the “washout period”. A seizure event occurring during the same risk or control window was counted as a separate event if it occurred > 24 h after the first. Age and calendar season were assessed as potential confounders in the Poisson regression models. Statistical analysis was conducted using SAS version 9.4 (SAS Institute, Cary, NC).

## Results

**Fig. 1:**
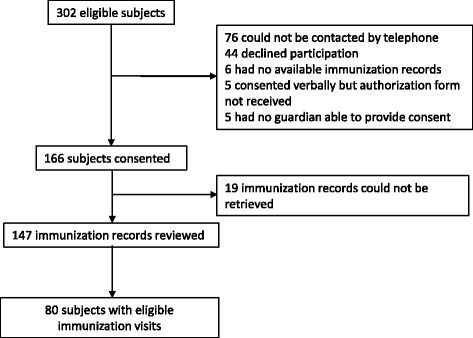
Summary of eligible subjects who did and did not provide consent to release their immunization records. Eligible immunization visits include immunizations administered between the date of the epilepsy diagnosis and the seventh birthday

In total, 302 children met the inclusion criteria, of whom 38% had partial (focal) epilepsy, 33% had unclassified epilepsy, 18% had idiopathic generalized epilepsy (52% of whom had absence epilepsy), 5% had benign childhood epilepsy with centrotemporal spikes (benign rolandic epilepsy), and 4% had severe epilepsy. Parents of 166 children consented to release their immunization records (Fig. 1). Immunization information was available on 147 children from either Public Health or the primary care physician. Eighty children had one or more immunization visits between their epilepsy diagnosis and 7th birthday. Demographic and clinical characteristics are shown in Table 1. Children with immunization events were diagnosed at a younger mean age (2.1 years versus 4.0 years versus 3.1 years, *p* < 0.001) and had more seizure events during the observation period (mean events per subject = 2.5 versus 0.7 versus 0.9, p < 0.001) than either children with no immunization events or children whose records were unavailable.

**Table 1:** Characteristics of children under 7 years of age with epilepsy seen between 2010 and 2014 (*N* = 302)

Characteristics	Patients with immunization visits (*N* = 80)	Patients with no immunization visits (*N* = 67)	Patients with no immunization records (*N* = 155)	*P*
	n (%)	n (%)	n (%)	
Sex				
Male	37 (46)	33 (49)	76 (49)	0. 93
Female	43 (54)	34 (51)	79 (51)	
Age at epilepsy diagnosis				
mean (SD), in years	2.1 (1.6)	4.0 (2.1)	3.1 (2.1)	< 0.001
Place of residence				
Halifax Regional Municipality	29 (36)	22 (33)	63 (41)	0.34
Outside Halifax	51 (64)	45 (67)	92 (59)	
Epilepsy type				0.06
Severe epilepsy	7 (9)	5 (7)	3 (2)	
Partial epilepsy	33 (41)	19 (28)	62 (40)	
Unclassified epilepsy	26 (32)	21 (31)	52 (34)	
Other types	14 (18)	22 (33)	38 (25)	
Number of seizure events				
mean (SD)	2.5 (3.2)	0.7 (1.5)	0.9 (1.6)	< 0.001
Antiepileptic medications				0.01
0	6 (7)	9 (13)	36 (23)	
1	55 (69)	45 (67)	99 (64)	
≥ 2	19 (24)	13 (19)	2 (13)	

CI, confidence interval; SD, standard deviation

There were 161 immunization events among the 80 subjects who received immunizations between their epilepsy diagnosis and seventh birthday (Table 2). Thirty-nine of 59 (67%) children with immunization records who were diagnosed with epilepsy before 4 years of age and followed until ≥6 years of age had ≥1 immunization visit. Inactivated vaccines were administered at nearly all immunization visits, either alone or with live vaccines.

**Table 2:** Immunizations administered after epilepsy diagnosis

	N	%
Number of immunization visits per subject (N = 80)		
1	45	31
≥ 2	35	24
Vaccine type(s) administered at immunization visits (*N* = 161)		
Inactivated vaccine(s) only	94	58
Live vaccine(s) only	7	4
Live and inactivated vaccines co-administered	60	37
DTaP	76	47
Influenza	27	17
MenC	7	4
MMR/MMRV	22	14
PCV	16	10
Rotavirus	3	2
Varicella	5	3
Other	5	3
Age at immunization event (N = 161)		
0–11 months	39	24
12–23 months	47	29
24–47 months	21	13
48–83 months	54	34
Season of immunization (N = 161)		
Winter	30	19
Spring	30	19
Summer	56	35
Fall	45	28

DTaP, Diphtheria-Tetanus-acellular Pertussis; MenC, meningococcal conjugate vaccine; MMR, measles-mumps-rubella; MMRV, measles-mumps-rubella-varicella; PCV, pneumococcal conjugate vaccine

Children with immunization visits had a total of 197 healthcare encounters for seizure during the study period. The majority of encounters were telephone calls to the neurologist (131/197; 66%), followed by ED visits (31/197; 16%), hospitalizations (20/197; 10%), and unscheduled outpatient visits (15/197; 8%). The number of seizure events was similar across all four seasons, ranging from 54 events in winter (27%) to 43 events in summer (22%). The mean age at the time of the seizure event was 3.5 years (standard deviation = 1.7).

The results of the risk interval analysis are shown in Table 3. The relative risk of seizure was not increased during the risk period 0–14 days after any vaccine versus the control period 21–83 days post-immunization (RR = 1.14, 95% CI: 0.46, 2.83). Similarly, there was no increase in the relative risk of seizure events 0–2 days versus 21–83 days after inactivated vaccines. No seizure events occurred during the risk periods 7–10 days or 5–14 days after a live vaccine; therefore the RR could not be estimated. In sensitivity analyses, the RR was estimated using a 0–3 day risk interval after inactivated vaccines, and a control period of 7–83 days prior to immunization (see Additional file 1). In all models, the estimate of RR was similar to the primary analysis.

**Table 3:** Risk interval analysis among children with epilepsy and immunization events (*N* = 80)

Vaccine type	Healthcare encounters for seizure after immunization					RR^a^
	Risk period				Control period	
	0–14 days	0–2 days	7–10 days	5–14 days	21–83 days	(95% CI)
Any type	6	–	–	–	20	1.14 (0.46, 2.83)
Inactivated	–	1	–	–	20	0.95 (0.13, 7.06)
Live	–	–	0	0	10	-^b^

RR, relative risk

^a^Including age or calendar season in the model did not change the estimate of RR, therefore the unadjusted models are presented

^b^ Unable to estimate RR

The rate of seizure events during the 0–14 day risk period after any immunization was 0.92 events per person-year versus 0.76 events per person-year during the control period, which corresponds to a rate difference of 0.16 seizure events per person-year (95% CI: -0.67, 0.98). The upper confidence limit corresponds to 1 additional seizure event in the 0–14 day risk period per 25 immunizations. The rate of seizure events was 0.790 per person-year during the 0–2 day risk period versus 0.788 per person-year in the control period, corresponding to a rate difference of 0.002 seizures per person-year (95% CI: -1.611, 1.615). The upper confidence limit corresponds to 1 additional seizure event in the 0–2 day risk period per 75 inactivated immunizations.

## Discussion

In this study, children < 7 years of age who were followed by a neurologist for epilepsy did not appear to be at increased risk of seizure requiring medical attention after any immunization or after inactivated vaccines, compared to their baseline risk. The lack of seizure events during the risk periods after live vaccines suggests that the risk of seizure is not increased after live vaccines, although the relative risk could not be estimated.

The study findings are consistent with a self-controlled case series of adults and children 0–24 years of age with and without epilepsy which found no increased risk of seizure requiring inpatient or outpatient hospital care during the risk periods 0–7 days or 8–30 days after adjuvanted pandemic H1N1 influenza immunization versus the controls periods 31–90 days pre-immunization or 31–90 days post-immunization. [20] However, the risk was not assessed specifically in young children and the 0–7 day risk period may have been too long to detect a transient increase in risk 24–48 h after an inactivated vaccine. [7, 8] In addition, this study captured only seizure events severe enough to require hospital care, while our study also captured non-severe seizure events managed via telephone or in the outpatient clinic.

In contrast, a study of 17 children with severe myoclonic epilepsy of infancy whose parents completed seizure diaries after MMR immunization reported an incidence rate ratio of parent-reported seizure of 2.3 (95% CI: 1.5–3.4) in the 5–12 days after the first MMR dose versus the control periods of 0–4 days and 13–42 days post-immunization. [21] Children with severe myoclonic epilepsy of infancy are particularly susceptible to fever-induced seizure, which may explain the increased risk of seizures after MMR vaccine during the period when MMR-associated febrile seizures are observed. In addition, in 16% to 21% of children with severe myoclonic epilepsy of infancy, their first seizure occurred after immunization. [21, 22] Only 15 children in our study had a severe epilepsy syndrome (severe myoclonic epilepsy of infancy, infantile spasms, or Lennox-Gastaux syndrome); therefore, we are unable to exclude a risk in this subgroup. While we may have expected to see an increased risk of seizures after MMR/MMRV in this study, most participants were diagnosed with epilepsy after 12 months of age when the first dose is recommended. The second MMR dose does not appear to be associated with an increased risk of febrile seizure. [23] Further evaluation of vaccine safety in these high-risk children is needed.

Although not an objective of our study, our results provide some evidence to suggest that children with epilepsy may be underimmunized for their age. Only 17% of children with immunization records had received influenza vaccine which is recommended annually for all children over 6 months of age, especially those with epilepsy who are at increased risk of severe influenza. [24] Twenty of 147 (14%) children whose immunization records were available had no recorded immunization events between ages 4 and 7 years, suggesting that they had not received their preschool booster immunizations. It is not clear whether these children were less likely to be immunized than children without epilepsy as overall vaccine coverage in Nova Scotia is below target levels. [25] However, limited evidence suggests that children with epilepsy are less likely to be fully immunized than children without epilepsy. [26, 27] Whether a diagnosis of epilepsy negatively impacts future vaccine uptake is an important question that merits further study.

This study had limitations. We were unable to obtain immunization records on all patients and records may have been incomplete, underlining the importance of central immunization registries for evaluating vaccine safety. Children with immunization events experienced more seizure events than those without immunization events or whose records were not available, which would be expected to lead to an overestimation of the risk of post-immunization seizure. Ascertainment of healthcare encounters for seizure may have been incomplete if some telephone calls to the neurologist were not documented or if some emergency visits and admissions to hospitals outside of the IWK were not reported to the neurologist. In addition, if parents were warned that seizures could occur after immunization, they may have been less likely to report post-immunization seizures. This could have led to an underestimation of the risk of post-immunization seizure. The precision of the results was limited by the small sample size and there were no healthcare encounters during the risk period after a live vaccine, which precluded determination of the relative risk. Nonetheless, we demonstrated that the upper limit of the attributable risk of seizure during the 0–14 day and 0–2 day risk intervals was only 1 seizure per 25 immunizations and 1 per 75 inactivated immunizations, respectively, with two-thirds of events being mild enough to be managed by telephone. A strength of this study was the ascertainment of both severe and non-severe seizure events after immunization among children with all types of epilepsy.

## Conclusions

Parents and vaccine providers can be reassured that children with epilepsy do not appear to be at increased risk of medically attended seizure after immunization. While a small increased risk of seizure after immunization cannot be excluded, when balanced against the risk of seizure associated with a vaccine-preventable infection, the benefits appear to outweigh the risks. Further studies are needed in different populations to confirm these findings.

## Additional file

Additional file 1: Results of sensitivity analyses of risk interval analysis. This table presents the results of the analysis of the risk of seizure 0–14 days after any vaccine versus 7–83 days before immunization, the risk of seizure 0–2 days after an inactivated vaccine versus 7–83 days before immunization and the risk of seizure 0–3 days after an inactivated vaccine versus 21–83 days post-immunization. (XLSX 9 kb)

## Abbreviations

AEFI: Adverse event following immunization
CI: Confidence interval
ED: Emergency department
MMR: Measles-mumps-rubella vaccine
RR: Relative risk
